# Intraoperative technologies to assess margin status during radical prostatectomy – a narrative review

**DOI:** 10.1038/s41391-024-00868-2

**Published:** 2024-07-18

**Authors:** O. Windisch, M. Diana, D. Tilki, G. Marra, A. Martini, M. Valerio

**Affiliations:** 1 Service of Urology, Department of Surgery, Geneva University Hospitals, Genève, Switzerland; 2 Faculty of Medicine, Geneva University, Genève, Switzerland; 3 Martini-Klinik Prostate Cancer Center, University Hospital Hamburg Eppendorf, Hamburg, Germany; 4 Department of Urology, University Hospital Hamburg-Eppendorf, Hamburg, Germany; 5 Department of Urology, Koc University Hospital, Istanbul, Turkey; 6 Department of Surgical Sciences, San Giovanni Battista Hospital and University of Turin, Turin, Italy; 7 Department of Urology, University of Texas MD Anderson Cancer Center, Houston, TX USA

**Keywords:** Prostate cancer, Cancer therapy

## Abstract

Positive surgical margin (PSM) is a frequent concern for surgeons performing radical prostatectomy for prostate cancer (PCa). PSM are recognized as risk factors for earlier biochemical recurrence and expose patients to adjuvant or salvage treatments such as external radiotherapy and hormonotherapy. Several strategies have been established to reduce PSM rate, while still allowing safe nerve-sparing surgery. Precise preoperative staging by multiparametric magnetic resonance imaging (mpMRI) and fusion biopsy is recommended to identify suspicious areas of extracapsular extension (ECE) that warrant special attention during dissection. However, even with optimal imaging, ECE can be missed, some cancers are not well defined or visible, and capsular incision during surgery remains an issue. Hence, intraoperative frozen section techniques, such as the neurovascular structure-adjacent frozen section examination (NeuroSAFE) have been developed and lately widely disseminated. The NeuroSAFE technique reduces PSM rate while allowing higher rate of nerve-sparing surgery. However, its use is limited to high volume or expert center because of its high barrier-to-entry in terms of logistics, human resources and expertise, as well as cost. Also, NeuroSAFE is a time-consuming process, even in expert hands. To address these issues, several technologies have been developed for an ex vivo and in vivo use. Ex vivo technology such as fluorescent confocal microscopy and intraoperative PET-CT require the extraction of the specimen for preparation, and digital images acquisition. In vivo technology, such as augmented reality based on mpMRI images and PSMA-fluorescent guided surgery have the advantage to provide an intracorporeal analysis of the completeness of the resection. The current manuscript provides a narrative review of established techniques, and details several new and promising techniques for intraoperative PSM assessment.

## Introduction

Positive surgical margin (PSM) is a frequent surgeon concern, that has been reported to occur between 14–24% of the cases in high-volume center with experts surgeons, while higher number have been reported for locally advanced disease or less expert centers [1, 2]. Neurovascular bundle (NVB) sparing surgery is recognized as a safe procedure in the hand of expert surgeon in well-selected patient, but has been associated with increased rate of PSM in less experts surgeons during their learning curve [3–6] While extensive and multifocal PSM were long demonstrated as associated with increased BCR and successive or salvage treatments, recent evidence showed that limited (< 3 mm) PSM were also significantly associated with increased BCR [7, 8] Evidence evaluating patient anxiety after PSM announcement is lacking, but incomplete resection can be a stressful experience for patients, and a challenge to face for surgeons.

Several locations are more prone to PSM on the radical prostatectomy (RP) specimen; the most frequent site being the apex and the second one the posterolateral margins of the prostate where the NVB lie. The main reason for this distribution is related to functional considerations. At the apex, the pseudocapsule ends in a diffuse manner limiting the precise ability to identify its distal end, while the surgeon tries to preserve the maximal functional urethral length. At the posterolateral margin, PSM are mainly observed in case of advanced nerve-sparing surgery or when ECE is present. Other margins location exist (bladder neck, prostate capsule, seminal vesicles) but are less common [9].

To better plan nerve-sparing surgery approach (intrafascial, interfascial or extrafascial plane), risk and location of ECE has to be assessed. Several preoperative strategies have been developed, frequently based on preoperative imaging or nomograms to predict zonal ECE. In Europe, multiparametric magnetic resonance imaging (mpMRI) is recommended in the diagnostic pathway prior to biopsies and it can help identifying ECE with several distinctive signs (i.e. broad contact of the tumor with the capsule, tumor in periprostatic fat etc.) [10]. Other imaging techniques, such as PSMA-PET or micro-ultrasound have also been reported but lack sufficient evidence in the context of PSM assessment [11, 12]. Several nomograms were also developed to evaluate the risk of side-specific extraprostatic extension [13].

This is a dilemma in the intraoperative decision-making in which the surgeon must compromise between the functional and the oncological outcome. If an intraoperative assessment of surgical margin was available, the surgeon could push the boundaries of NVB preservation, and at the same time ensure negative margins [14]. As high as 80% surgeons reported in a recent survey that they would find useful to have intraoperative technologies to assess for PSM [15]. Due to the low performance of unsystematic, cognitive-guided intraoperative frozen section, more systematic techniques have been developed to provide intraoperative assessment of surgical margins ; the most commonly reported being the neurovascular bundle structure-adjacent frozen section examination (NeuroSAFE). While very useful and accurate, the NeuroSAFE requires dedicated expert pathologist, and expensive resources, limiting its widespread use [16, 17].

Several novel technologies have been developed and reported - in vivo (intracorporeally during the intervention), such as augmented reality with 3-D reconstruction of mpMRI images, or PSMA-fluorescence guided surgery, - or ex vivo (the specimen is extracted from the body and analyzed), such as fluorescent confocal microscopy (FCM) and specimen PET-CT. The current manuscript begins with a narrative review of established techniques, and details new and promising technologies in its second part.

## Methods

This non-systematic review was performed searching on the Medical Literature Analysis and Retrieval System Online (MEDLINE), Web of Science, and Google scholar using a combination of the following keywords: “frozen section”,“intraoperative”,”real time”,”prostatectomy”,” margin”,“surgical margin”,”technology”,”imaging”,”fluorescence” on December 15th 2023. Furthermore, manual search in the references of the included articles was performed and additional articles deemed relevant were included. The authors have not included all technologies in this non-systematic review, but focused their search on the technologies deemed to be used frequently, have an historical importance, or believed to have a wider use in the near future. The authors decided to stratify modern technologies according to preoperative, intraoperative in vivo or intraoperative ex vivo approach to assess surgical margins. Figures were created with Biorender.com.

### Preoperative technologies

In the present section, the authors focus on the technologies that are used preoperatively to assess the location(s), extension, and the risk for ECE. These technologies do not provide intraoperative feedback to evaluate the completeness of resection but play a role on the surgical planning.

#### Imaging techniques to improve staging accuracy and detect extraprostatic extension

mpMRI is recommended by the European Association of Urology guidelines within the prostate cancer diagnostic pathway and is therefore widely available before surgery. Despite being generally employed to guide surgery, a 2016 meta-analysis on 75 studies showed that the overall sensitivity for ECE was low (0.57) while the specificity (0.91) was high [18]. The low sensitivity resulted in additional recommendations to improve the quality of image acquisition and interpretation, such as the PI-RADS v2.1 revision and the PI-QUAL score, that aims to objectively assess mpMRI quality. When including only the best imaging quality and employing state-of-the-art ECE radiologic criteria, increased sensitivity was reported ; some authors reported that negative mpMRI could completely rule out ECE, while others reported that up to 20% patients still harbor missed ECE on the RP specimen [19, 20]. When comparing mpMRI to non-mpMRI diagnostic pathway, conflicting results have been reported, some authors claiming that reduced PSM and higher cancer control can be achieved, while others found similar PSM not allowing to draw definite conclusion [21–23]. No matter the final clinical impact, 26% to 59% of the clinicians considered that mpMRI had an impact on the surgical decision [24].

Microultrasound, a novel technology using a 29-MHz transrectal probe, has been increasingly adopted to improve identification and characterization of intraprostatic PCa significant lesions [12]. Its diagnostic ability, when compared to conventional TRUS that usually performed badly, have been described as superior with a sensitivity, specificity, PPV and NPV of 68.7, 96.3%, 80.8% and 93.1% respectively for the index lesion identification. It has already been used to assess and predict ECE, with a sensitivity of 72.1% and specificity of 88% resulting in an AUC of 80.4 [25]. It has not yet been employed in a prospective study to influence NVB preservation.

PSMA-PET-CT is mainly used for nodal or metastatic burden assessment. PSMA PET-CT underperformed mpMRI in detecting ECE in several studies [11, 26, 27]. However, PSMA-PET-MRI could represent an interesting alternative with a higher T-staging accuracy than mpMRI alone, with a global accuracy of 82.5%, a T3a accuracy of 79% and T3b of 94% [28]. While these results are interesting, the access to such imaging modality is highly variable depending on different countries. While the spatial resolution is best with mpMRI, a recent meta-analysis suggested that PSMA PET-CT is very promising, and that PSMA-PET-MRI shows a better sensitivity and specificity for ECE detection than conventional mpMRI [29].

#### Nomograms to predict extracapsular extension

Several prediction models have been described and further assessed in external validation studies, using several common variables (clinical T-stage, PSA, cancer burden using different metrics on biopsy etc.) and most of them including imaging-derived variables. While these nomograms perform adequately on the base population they were developed for, most of them present with significantly lesser accuracy at external validation. Two external validations studies of the promising models were published in 2019 and 2023. The best performing models (Patel, Pak, Martini, Soeterik) had an AUC ranging from 0.73 to 0.77 [13, 30]. While some authors developed interface-friendly scores to use (www.prece.it) to face the challenge of applying nomograms in real-life, the accuracy of even the best performing model still remains imperfect. Thus, nomograms provide further guidance in the nerve-sparing decision but are often not sufficient ; they ask for more precise technologies that are more adapted to each individual patient [31].

### Intraoperative strategies

Intraoperative strategies provide immediate feedback with regard to the completeness of resection, and an opportunity for additional resection in case of residual tumor detection. Ideal new technologies should be usable in vivo, be fast to use (minutes), in real-time, easy, provide an assessment of the entire surface of the prostate, whilst maintaining a high accuracy and without damaging the specimen for successive conventional histopathological examination [32]. This section will focus on modern “ex vivo” and “in vivo” technologies already adopted in clinical use as well as undergoing clinical implementation for intraoperative surgical margin assessment.

#### Ex vivo (extraction of the specimen required)

The technologies that are performed ex vivo rely on the previous extraction of the specimen. Whilst the ex-vivo analysis is ongoing the surgeon can either wait the results or finish the RP. Hence, timing required for the analysis are crucial. Extracting the specimen is a short operation when using appropriate surgical techniques with a very low risk of incisional hernia (< 1%) [33]. Ex vivo analysis allows for the detection of PSM, but does not allow an immediate in vivo visualization of where the suspicious residual tumor may lie. Orientation of the specimen, as well as the corresponding NVB has to be performed to guide the additional resection, but no standardized technique has been established since several techniques and technology co-exist. In addition, the objective of re-resection to obtain negative surgical margins is believed to be a safe procedure and bring oncological benefits, but these oncological benefits have not yet been proven [34].

#### Intraoperative frozen section (Gold-standard)

**Fig. 1:**
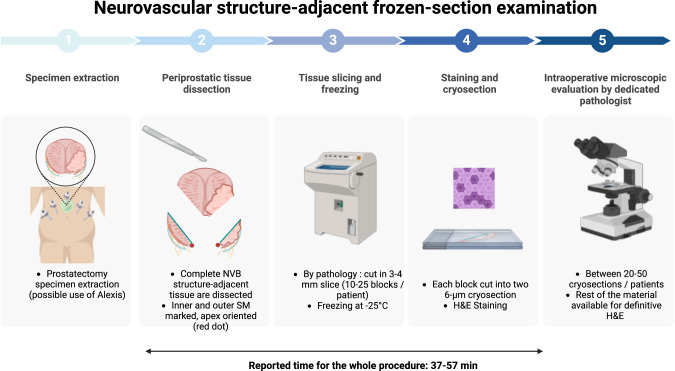
Details the whole process of the Neurovascular structure-adjacent frozen-section examination (NeuroSAFE) technique. H&E Hematoxylin & Eosin.

Intraoperative frozen section (FS) is the most frequently used procedure to assess surgical margins and is considered the gold-standard technique [24]. After freezing the specimen at a −16° to −20 °C, it is cut in multiple sections that are then stained using Hematoxylin and Eosin for microscopic evaluation. Since assessing the whole circumference of the specimen is time-consuming, and the procedure has to be performed intraoperatively without delaying too much the advance of the surgery, only specific areas of the gland at risk of PSM are examined. A recent systematic review provides a list of the recently published studies that reported FS during prostatectomy [35]. It is interesting to note that initially, no standardized techniques were used, and that some authors performed FS only on the apical site, or neurovascular bundle, bladder neck and sometimes a combination of these [24]. In 2012, Schlomm reported the standardized technique of neurovascular structure-adjacent frozen examination (NeuroSAFE) that assessed periprostatic tissue, with an accuracy of 97.3%, reducing the PSM rate by a 32% rate (from 22% to 15%) while increasing the ability to provide nerve sparing [36]. Figure 1 describes the NeuroSAFE technique. Since then, several other studies have reported promising results of the NeuroSAFE [35]. To translate this number into clinically meaningful outcomes for patients, a randomized control trial (NEUROSAFE PROOF) is now ongoing, comparing NEUROSAFE against standard of care in terms of recovery of potency after surgery (defined as an IIEF-5 ≥ 22) [17]. While valuable, these techniques require expert and available pathology teams, as well as a whole logistics that reduces greatly the number of centers worldwide able to adopt this technique as a standard-of-care.

#### Techniques based on intraoperative imaging

Several techniques have been developed to reduce the barriers of intraoperative FS. The main benefit of these techniques is that the preparation does not require a long freezing, fixing and staining process; the analysis is made simpler and quicker to perform than conventional FS and require reduced human and logistics resources. Some recent narrative and systematic review discussed some of the presented techniques [24, 32, 37]. Since these last reviews, more publications have described the role of ex vivo fluorescence confocal microscopy, intraoperative PET-CT and intraoperative fluorescence. and very recent studies showed the role of the PET-CT specimen imager (Aura-10, XEOS, Belgium) as a promising tool for whole-mount specimen quick analysis [38, 39]. The following section details clinically available tools.

**Fig. 2:**
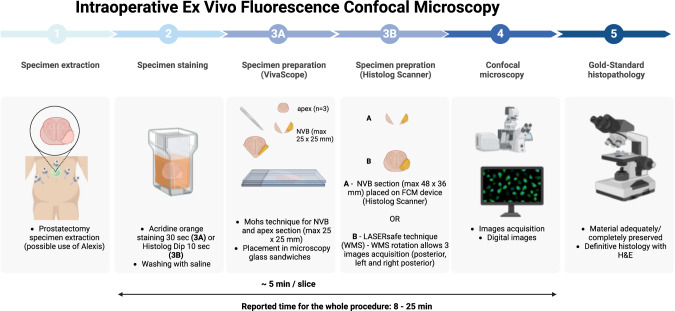
Details the whole process of ex vivo fluorescence confocal microscopy from specimen extraction to confocal microscopy. It has to be noted that the material is adequately or completely preserved for definitive H&E analysis depending on the device used. H&E Hematoxylin & Eosin.

Fluorescence confocal microscopy (FCM) was first described in 2019 by Pulia, on prostate biopsies obtained from a RP specimen. Biopsies were prepared by a staining process (immersion in an acridine orange solution for 30 s and then rinsed with physiological saline) and then embedded between two glass slides fixed with glue. FCM acquisition was then performed, and acquired digital images were compared to gold-standard histopathological Hematoxylin & Eosin (H&E) analysis. They showed an 83.3% sensitivity and 93.5% specificity of FCM resulting in a 0.88 area under the curve and a high level of agreement (kappa coefficient of 0.75) [40]. The device employed (Vivascope 2500 M-G4; Caliber Imaging and Diagnostics, Rochester, NY, USA) uses two different lasers that enable examination according to reflectance (785 nm) and fluorescence (488 nm). It has a vertical resolution of 4 um and maximum depth examination of 200 um, with a magnification of up to 550x. A total area of 25 x 25 mm^2^ can be acquired in the same image, explaining that several images may be needed to cover the whole gland. After this initial promising experience, Rocco et al reported the use of FCM for prostate biopsies on 54 patients, examining 854 images (427 glass slides and 427 FCM digital images) by 4 expert pathologists with the same process. The results were impressive with a 95.1% accuracy and an almost perfect interreader agreement for cancer detection although the agreement was moderate regarding cancer grade [41]. Marenco and colleagues reported similar results on prostate biopsy cores with an 85% positive predictive value (PPV) and 95.1% negative predictive value (NPV) in a median processing and analysis median of 5 min [42]. After the proof of concept, Rocco et al conducted a study on 20 patients with whole-mount specimen and showed a a perfect agreement between benign and cancerous glands and almost perfect for periprostatic tissue [43]. The same team described the use of the Mohs section to perform a flat cut of the neurovascular bundle that doesn’t require previous freezing of the specimen Using this technique, 8 patients had an intraoperative analysis of their surgical margins with a total duration of less than 25 min. All underwent bilateral complete nerve-sparing and one suspicion of PSM had a re-resection, that finally showed no residual tumor [44]. They later reported a standardized surgical technique; during dissection, clips are placed on the specimen and the neurovascular bundle at the same level for orientation and to guide potential re-resection. 3 slices are obtained from the apex using Mohs technique, and from the posterolateral right and left neurovascular bundle. The rest of process is summarized in Fig. 2. Digitalization of images offer the possibility for remote pathologist interpretation [45]. Using the previously described technique, but with a different device (Histolog Scanner, SamanTree Medical SA, Lausanne, Switzerland), with a scanning area of 48 x 36 mm^2^ Baas et al compared the performance of intraoperative ex vivo FCM with NeuroSAFE and showed a sensitivity, specificity, PPV and NPV of 86%, 96%, 80%, and 98% respectively. Both techniques found all relevant PSM (defined as residual tumor in the re-resected NVB), while the FCM procedure was significantly shorter (8 vs 50 min) [46]. Also, other authors reported the LASERfree technique that does not require any tissue cutting, allowing image acquisition by only applying and rotating the prostate posterior and postero-lateral side, to keep the specimen intact for further analysis [47].

**Fig. 3:**
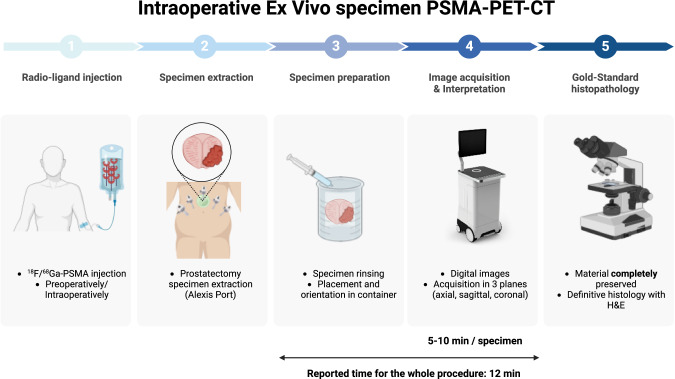
Details the whole process of ex vivo PET-CT specimen from radio-ligand injection to the PET digital images acquisition. It has to be noted that the material is fully preserved for definitive H&E analysis. H&E Hematoxylin & Eosin.

PET-CT specimen imager (Aura 10, XEOS, Belgium) is a technology using a PSMA ligand coupled with a radionuclide (either ^18^F or ^68^Ga) that is injected intravenously, as for PET-PSMA. The injection may be performed before the intervention, with the acquisition of a conventional whole-body irradiation (whole body PET-CT) to assess for positive lymph node or lesions that would contra-indicate the intervention. After the injection, the prostatectomy specimen is retrieved (as well as lymph nodes if indicated) and extracted through an Alexis^TM^ port. The specimen is then rinsed from any residual blood clot and oriented in a specific container. Afterwards, digital images in 3 planes (axial, sagittal and coronal) are acquired and interpreted; images from the PET-CT are superimposed to the CT-scan to allow for margin inspection, at an inframillimetric resolution. Figure 3 details this process. This process was first reported by Darr and al. in 2023 and showed the feasibility of this technique to assess prostatectomy specimen for PSM, as well as metastasis in lymph nodes, on 10 consecutive patients that presented with locally advanced prostate cancer, with 6 patients receiving ^68^Ga-PSMA-11 and 4 receiving ^18^F-PSMA-1007. The device consistently detected the index lesion previously identified on mpMRI and had a 93.5% correlation rate with PET-CT, while the only missed lesion was negative on final pathology, suggesting a possible contamination of the PET-CT by the urine. The resolution was estimated to be 1 mm for the PET and 0.2 mm for the CT, with final superimposed images acquired in a median time of 12 min. It allowed the detection of 4 PSM in pT3 stage patient, that were later confirmed on final histology [38]. Oderda et al. also reported to use of the Aura-10 on 3 patients presenting with locally advanced disease operated on for RP and extended pelvic lymph node dissection. The specimen-CT correctly identified a positive lymph nodes, previously seen on whole-body PET-CT, and confirmed the absence of PSM in 2 specimen while the third was uncertain with a final pathological report confirming a complete resection. Median time for image acquisition was also 12 min [39]. The main issue using this technology is the additional irradiation for the patient, as well as for the assistant surgeon, scrub nurse and surgeon. These issue were assessed previously by the same team, using the same radioligand with another imaging technology (Cerenkov Luminescence), where they showed an acceptable irradiation rate for exposed health professionals, 9.0 μSv (±7.1) for the first assistant, 3.3 μSv (±3.9) for the scrub nurse, 0.7 μSv (±0.7) for the imaging-device management or surgeon [48, 49]. The technology is still new, despite having being used in other indications as well (breast, head and neck, pancreatic adenocarcinoma, neuroendocrine tumors). It still requires a standardization on how the signal should be interpreted (SUV range), the optimal time between injection and interpretation, the best radiotracer to use (Gallium showed a better noise-to-background but lesser resolution, while Fluor has a longer half-life that may be useful depending on when the patient should receive the injection). To address this issue, Rodeva and al developed a machine learning algorithm to provide an automatic 3D-segmentation of PET-CT specimen in 6 specimens with a high weighted average precision (97–99%), suggesting that the manual segmentation that was performed manually in the previously reported study by Darr (to compare concordance of the SUV signal with definite histology), may be done automatically in the future, allowing for a larger role of machine learning [50].

#### In vivo (Prostatectomy specimen left in place, analysis done intracorporeally)

In vivo analysis has been reported using several technologies such as augmented reality based on mpMRI, as well as fluorescent confocal endomicroscopy. The most recent development in in vivo technology relies on fluorescent probes. To define a successfully developed fluorescent probe, Mandel stated the four following tenets; 1. high specificity and good signal-to-noise ratio, 2. feasibility of real time imaging lasting during the whole procedure, 3. biosafety and low toxicity profile/adverse effects, 4. limited cost allowing for widespread use. When using fluorescence, the Near-InfraRed (NIR) spectrum has the advantage not to hinder normal vision under white light since its spectrum lies outside of visible light [51]. The main benefit of an intraoperative in vivo assessment is an easier detection of where the residual tumoral tissue lies, allowing for a targeted additional resection [52]. Holding this into account, the current section will detail “in vivo” technologies.

##### MpMRI combined with augmented reality

Several authors showed the feasibility of superimposing 3-D prostate models obtained from previous radiological images (TRUS, mpMRI) as augmented reality models on intraoperative field, allowing a significant reduction in PSM rate [37, 53, 54]. The main problem encountered with both imaging modalities is the underestimation of the tumor size on the 3-D modeled prostate and tumors (3-D model tumor volume represented 48% and 82% of the real volume for TRUS and mpMRI respectively). This resulted in the need for better 3-D superimposition that was later described by Porpiglia, by integrating the reconstruction directly in the robotic console through the TilePro option. This allowed the authors to proceed to AR-guided biopsies that confirmed extracapsular location in cT3 patients in 78% of the biopsies [55]. After this promising experience, they incorporated elastic fusion in their reconstructed model, allowing to take into account the tissue deformation occurring during the intervention. With this system, they identified 100% of the extracapsular involvement in 20 patients presenting with cT3 disease, compared to a 47% accuracy of a 2D cognitive guided resection [56]. Another simple form of augmented reality is the superimposition of an orientable 3-D model, reconstructed by a previous contouring of the prostate and lesions, that can be then oriented and integrated in the Da Vinci Console using the Tilepro function (Intuitive Surgical Inc., Sunnyvale, CA,USA) to better locate the tumor and guide the surgeons. This technique has been effective in reducing PSM (22.5% vs 11.3%) with 35% of the PSM located in mpMRI negative lesions [57].

##### Confocal laser endomicroscopy

Mainly reported ex vivo, fluorescence confocal microscopy has also been reported as an in vivo procedure by Lopez in 2016 in a 15 patients cohort. It allowed the assessment of selected prostatic capsule regions, as well as periprostatic tissues (bladder neck, urethral stump, levator ani and obturator nerve). The integration of the technology with the TilePro function of the Da Vinci surgical robot (Intuitive Surgical Inc., Sunnyvale, CA,USA) was described as easy to implement. This technology uses a fiberoptic probe (either 2.6- or 0.85 mm diameter), that contain laser bundles, allowing to obtain microscopic images at a 1000-fold magnification when putting the probe in contact of the tissue. As for ex vivo, a preparation of the tissue with fluorescein is required, with an intravenous injection 5 min prior to neurovascular bundle dissection [58]. To provide better interpretation of the findings, Panarello provided an Ex Vivo Atlas of prostatic and periprostatic tissues to simplify interpretation [59]. This technique is also widely used in gastroenterology and has also been used in urology for upper tract urinary cancer in urology. To our knowledge, this technology has not been reported afterwards for prostatectomy, but a clinical trial with unpublished results was launched in 2019 [60].

##### PSMA-guided near-infrared (NIR) fluorescence

**Fig. 4:**
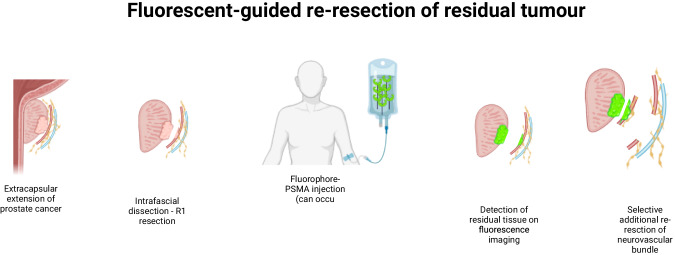
Provides a generic illustration of surgery guided by fluorescent with previous injection of PSMA-coupled fluorophore and possibility of targeted re-resection.

Mangano reported a first promising use of indocyanine green (ICG) to detect the prostatic artery location inside the NVB. It allowed the detection of 100% of the 62 assessed patients, but could not be directly used for margin assessment [61]. A step forward was made when Stibbe first reported in 2023, in a phase 2a trial, the use of OTL78, a PSMA- fluorescent tracer on 18 patients that underwent RP, using the VisionSense near-infrared imaging system (Medtronics, Minneapolis, MN, USA) intraoperatively to assess for cancer location and residual tumor detection after extraction of the specimen. Additional resection of suspicious areas were performed in 12 patients, and 3 of these patients had confirmed cancerous tissue on the re-resected areas. Interestingly, they also looked at time between injection and surgery and reported that all patients that had an injection 24 h before surgery and presented with suspicious residual fluorescence had confirmed residual cancer on the re-resection analysis. They showed a decreased performance of residual tissue detection when the injection was performed the same day compared to the previous day, and varying sensitivity depending on ISUP grade, tumor volume and PSMA expression [62]. Just after, Nguyen and al reported the use of IS-002, a near-infrared fluorescently labeled PSMA-targeting peptide, in a phase 1 trial. IS-002 was injected 24 h before surgery (based on preclinical pharmacokinetic and dynamic data) [52]. During RP with extended pelvic lymph node dissection, a fluorescence image-guided technology (Firefly, Intuitive Surgical Inc., Sunnyvale, CA,USA) was used to assess for residual tissue detection during and after specimen extraction, as well as for lymph node dissection. Surgeons had to assess on a Likert scale of 1–5 the degree of suspicion when analyzing the images. Using a cut-off of 3 defining “suspect”, they reported a 100% negative predictive value as well as an 80% positive predictive value of residual or locoregional tissue detection. The main advantage was, as previously seen with OTL78, a real-time assessment, with the detection of residual tissue, not detected by conventional white light, allowing for a guided re-resection as well. The principle of fluorescent-guided re-resection is illustrated in Fig. 4. The best performance was seen with the smallest dose of 25 ug/kg, since increasing doses, resulted in a higher signal intensity but background noise, reducing the positive predictive value and overall accuracy. These two examples provide promising example of selective fluorophores, with good intensity-to-background noise ratio, usable in clinical practice, with a safe administration profile [52]. Further research is required to assess the reproducibility of these techniques, to determine whether the re-resection improves the oncological outcomes, and if a functional impact can be observed by adopting this technology.

## Conclusion

PSM is a risk factor for earlier biochemical recurrence and one of the rare adjustable factors on which the surgeon can have an impact. While imaging and nomograms play an important role in identifying and predicting extracapsular disease, they have limitations and there is a need to further intraoperative decision-making. Intraoperative surgical margin with the NeuroSAFE technique is useful, reduces the rate of PSM and improves the rate of nerve sparing surgery, but has important barriers-to-entry limiting wide adoption.

Several promising technologies have been reported such as ex vivo confocal microscopy, PET-CT specimen imager to provide a whole prostate assessment allowing for whole specimen analysis in shorter time and with no need for dedicated in-room pathologists. Recently, PSMA-coupled fluorophores allowed for in vivo assessment of residual cancer during prostatectomy and additional resection. While their potential is clear to most clinicians, and the speed of their development impressive, their impact on functional outcome first, through patient reported-outcomes assessment, as well as solid oncological outcomes (biochemical recurrence, adjuvant treatment, mortality), following the IDEAL-D framework should be assessed before widespread adoption.
